# Breast Density Evaluation According to BI-RADS 5th Edition on Digital Breast Tomosynthesis: AI Automated Assessment Versus Human Visual Assessment

**DOI:** 10.3390/jpm13040609

**Published:** 2023-03-30

**Authors:** Daniele Ugo Tari, Rosalinda Santonastaso, Davide Raffaele De Lucia, Marika Santarsiere, Fabio Pinto

**Affiliations:** 1 Department of Diagnostic Senology District 12, Palazzo della Salute, Caserta Local Health Authority, 81100 Caserta, Italy; 2 Department of Economics, University of Campania “L. Vanvitelli”, 81043 Capua, Italy; 3 Department of Radiology, “A. Guerriero” Hospital, Caserta Local Health Authority, 81025 Marcianise, Italy

**Keywords:** breast cancer, breast density, screening, digital breast tomosynthesis, mammography, artificial intelligence, machine learning, Quantra, automated volumetric breast density software, visual assessment

## Abstract

Background: The assessment of breast density is one of the main goals of radiologists because the masking effect of dense fibroglandular tissue may affect the mammographic identification of lesions. The BI-RADS 5th Edition has revised the mammographic breast density categories, focusing on a qualitative evaluation rather than a quantitative one. Our purpose is to compare the concordance of the automatic classification of breast density with the visual assessment according to the latest available classification. Methods: A sample of 1075 digital breast tomosynthesis images from women aged between 40 and 86 years (58 ± 7.1) was retrospectively analyzed by three independent readers according to the BI-RADS 5th Edition. Automated breast density assessment was performed on digital breast tomosynthesis images with the Quantra software version 2.2.3. Interobserver agreement was assessed with kappa statistics. The distributions of breast density categories were compared and correlated with age. Results: The agreement on breast density categories was substantial to almost perfect between radiologists (κ = 0.63–0.83), moderate to substantial between radiologists and the Quantra software (κ = 0.44–0.78), and the consensus of radiologists and the Quantra software (κ = 0.60–0.77). Comparing the assessment for dense and non-dense breasts, the agreement was almost perfect in the screening age range without a statistically significant difference between concordant and discordant cases when compared by age. Conclusions: The categorization proposed by the Quantra software has shown a good agreement with the radiological evaluations, even though it did not completely reflect the visual assessment. Thus, clinical decisions regarding supplemental screening should be based on the radiologist’s perceived masking effect rather than the data produced exclusively by the Quantra software.

## 1. Introduction

Breast cancer (BC) is the second-leading cause of death in women, and breast density is universally recognized as one of the main risk factors for its development [1,2].

The term breast density exclusively refers to the amount of epithelial and stromal elements in a breast and should not be confused with consistency on an objective examination. The different parenchymal density patterns were first described by Leborgne in 1953 [3] and later classified by Wolfe in 1976 [4]. The radiographic appearance of the breasts varies due to these differences in tissue composition and the consequential different radiographic attenuation properties of fat, stroma, and epithelium [5]. In particular, the fat is radiographically lucent and appears dark on a mammogram, while the epithelium and stroma are radiographically dense and look light. The denser the breast, the more difficult it is to identify a lesion due to masking phenomena [6]. The relative risk of high breast density is much lower than that of other important risk factors such as age, family history, reproductive history, and genetic mutations [7]. However, since screening studies in the literature report that mammographically dense breasts are relatively common (about 50% of the screened population), density alone contributes much more to cancer risk [8]. Indeed, Digital Breast Tomosynthesis (DBT) has been introduced in current clinical practice because it represents one of the most powerful tools to overcome the limitations of standard full-field digital mammography (FFDM) in the detection of BC in high-density breasts. Indeed, in contrast to FFDM, DBT obtains multiple mammographic images, allowing for a three-dimensional reconstruction of the breast with a unique compression. By allowing radiologists to scroll through breasts slice-by-slice, DBT can further mitigate the masking effect of dense breasts and allow the visualization of small breast cancers, such as those that present as tiny opacities or architectural distortions [9].

Therefore, the assessment of breast density is one of the main goals of radiologists, since it could help identify the best BC screening strategies. In the United States, the notification of breast density has become mandatory, since women with dense breasts could benefit from shorter screening intervals [10]. Several methods have been proposed to evaluate mammographic breast density. The visual methods are based on radiologists’ subjective classification and include the Wolfe, Tabár, and Boyd methods, the Visual Analogue Scale, the Breast Imaging Reporting and Data Systems (BI-RADS), and the Royal Australian and New Zealand College of Radiologists (RANZCR) synoptic scale [11,12,13,14]. Among these, the BI-RADS recommendations from the American College of Radiology (ACR) are most widely used in clinical practice in many countries [15].

**Figure 1:**
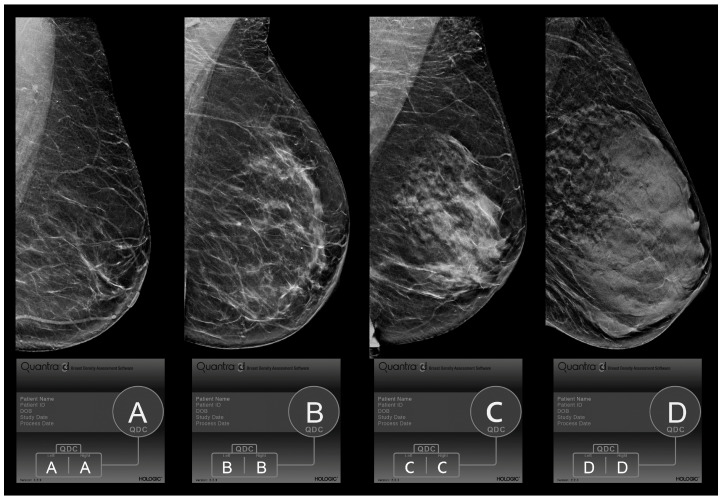
BI-RADS 5th Edition Quantra density categories (QDC). Evaluation of breast density categories performed by the Quantra softare (version 2.2.3) on digital breast tomosynthesis images according to BI-RADS 5th Edition. The lower boxes report the results of the Quantra evaluation for each breast and the final breast density assigned based on the denser breast. In these cases, since left and right breasts received the same evaluation, we showed only the left breast in medio-lateral-oblique (MLO) projection. Category A: almost entirely fat; category B: scattered fibroglandular densities; category C: heterogeneously dense; and category D: extremely dense.

The 4th edition of BI-RADS identified four categories based on the percentage of fibroglandular tissue present on the whole parenchymal area (category 1: <25%; category 2: 25–50%; category 3: 50–75%; category 4: >75%) [16]. The 5th edition of BI-RADS (Figure 1) [17] redefined this classification, giving more relevance to the qualitative analysis, so that even the presence of isolated areas of high breast density could increase the score due to the masking effect of a lesion. Specifically, by shifting the target from tissue percentage to parenchymal density, the new classification became more subjective, and the visual assessments of the BI-RADS categories showed a heterogeneous level of agreement between readers that varied from “slight” to “almost perfect”, as reported in previously published papers [18,19]. This discrepancy persisted due to many reasons, which included differences in the study populations, the reader’s level of experience, and the methods used in the study [20]. To support the analysis and to reduce the inter-reader variability, existing or new software has been upgraded to carry out an automatic assessment of breast density by analyzing mammographic images with machine-learning procedures, focusing on the distribution pattern of breast tissue rather than breast volumetric density alone.

Finally, Gastounioti A. et al. [15] showed an overall trend of decreasing breast density with a visual assessment when imaging was performed with DBT. To our knowledge, only one study [21] has evaluated the automated evaluation of breast density on DBT images. Consequently, the purpose of this study is to evaluate the concordance of the automatic classification of breast density performed on DBT images by the latest version (2.2.3) of the Quantra software (Hologic, Bedford, MA, USA) with the visual assessment, according to the BI-RADS 5th Edition criteria.

## 2. Materials and Methods

### 2.1. Study Design

**Figure 2:**
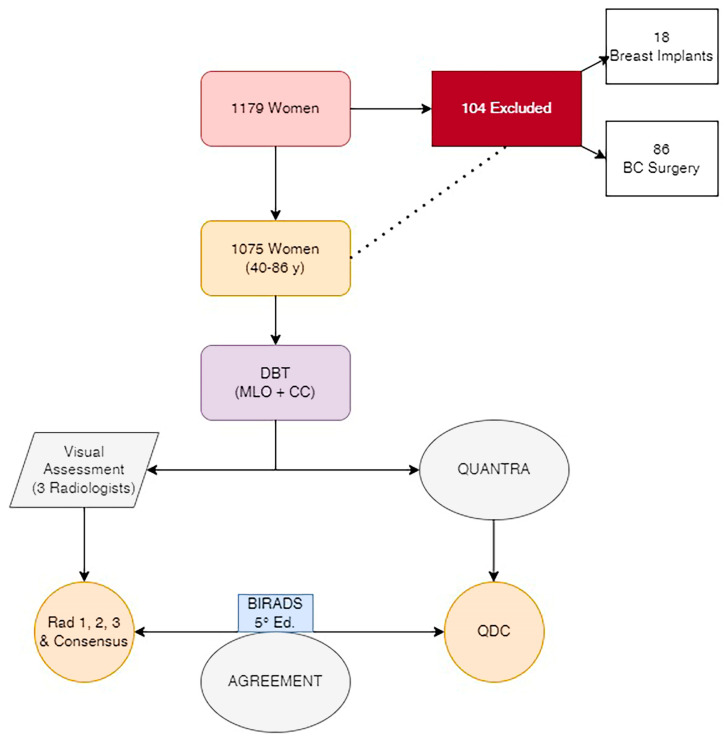
Flowchart. Study structure. DBT: Digital Breast Tomosynthesis. MLO: Medio-Lateral-Oblique projection. CC: Cranio-Caudal projection. Consensus: median value of the score assigned by the 3 radiologists. QDC: Quantra Density Category performed by the Quantra software.

A total of 1179 DBT examinations from women aged between 40 and 86 years old (58 ± 7.1), performed at our department between January 2022 and April 2022, were retrospectively analyzed in the study. Women with a history of breast surgery, breast augmentation, or chemotherapy were excluded. Routine mediolateral oblique (MLO) and craniocaudal (CC) views were obtained for each breast. DBTs have been performed with the Hologic Selenia Dimension system (Hologic, Bedford, MA, USA) with standard screening and automatic exposure control. The monitor settings and reading conditions remained unchanged throughout the study. One technologist with seven years of experience in mammography performed the exams to obtain similar compression techniques in all patients. The compression was complete when blanching occurred on the breast or the patient could not tolerate the pressure anymore. (Figure 2).

### 2.2. Visual Mammographic Density Assessment by Radiologists

According to the BI-RADS 5th Edition, three independent readers with different levels of experience analyzed mammographic densities. One reader was a breast radiologist with 10 years of experience in reading mammograms, and the other two were breast radiologists with 25 years of experience. The presentation of cases was randomized to reduce bias. Each reader was blinded to the assessment of the other radiologists and the automated breast density measurement.

Each acquired mammogram was assessed for breast density per breast (right or left), and per subject according to the BI-RADS breast density categories. For the assessment of breast density per subject, a BI-RADS breast density category was assigned based on the denser breast. After a review of the results from the three radiologists, a consensus was reached by discussion in cases of discrepancy in the BI-RADS breast density category of the mammogram.

BI-RADS assessment categories A and B were considered non-dense, and categories C and D were considered dense.

### 2.3. Automated Breast Density Assessment

Automated breast density assessment was performed by the Quantra software, version 2.2.3 (Hologic, Bedford, MA, USA). This is the latest version of software for the calculation of breast densities from both 2D digital mammographic and DBT images, with the help of DICOM for image processing. While a previous version of Quantra relied on volumetric breast density, version 2.2.3 provides an estimation of breast density category by analyzing the distribution and texture of the parenchymal tissue pattern. It is based on an AI algorithm derived from a machine-learning-based algorithm that uses a multiclass Support Vector Machine-based classification technique to segment breast types into four categories based on breast parenchymal tissue pattern and texture representation. A four-class SVM model was trained using over 6000 mammography studies for which the BI-RADS category assigned by radiologists at the time of screening was available as ground truth. This model focuses on the pattern and texture analysis of the mammographic image, and it is optimized to separate breast composition categories based on the distribution of tissue and the masking effect rather than volumetric measurement alone [22].

The Quantra 2.2 software will also have a user-selectable option to analyze either conventional 2D or tomosynthesis images in cases where both images are available for analysis when using the combined mode. When analyzing tomosynthesis images, the Quantra software uses a central projection image of the tomosynthesis acquisition. In our study, we used this latter method. For each mammographic study, a Quantra Breast Density Category (QDC) value was provided, representing the value for the denser breast and the QDC value for each breast.

### 2.4. Ethical Considerations and Data Availability

The study was conducted according to the guidelines of the Declaration of Helsinki. The Institutional Review Board is not applicable, considering the retrospective nature of the study. To publish this paper, informed consent was obtained from the patients before any radiological exam. The dataset can be found on saniarp.it, in the Caserta LHA reporting database, and in the register of our daily activities.

### 2.5. Statistical Analysis

Medical records were reviewed, and demographic data for age and personal history of breast augmentation, breast-conserving surgery, or mastectomy were compiled.

Weighted kappa values were calculated to analyze the proportion of inter-reader agreements in a visual density assessment. The kappa values were interpreted as follows: 0.0–0.20, slight agreement; 0.21–0.40, fair agreement; 0.41–0.60, moderate agreement; 0.61–0.80, substantial agreement; and 0.81–1.00, almost perfect agreement [23] (Table 1).

The analyses were also performed for the two broader categories (non-dense and dense) and for the age range (40–49 years old; 50–69 years old; and >70 years old). The distribution of breast density categories was compared by McNemar’s test, and *p* < 0.05 was considered statistically significant.

After the visual and volumetric assessments according to the BI-RADS category were compared, subjects were divided into either a concordant or discordant group, and the differences between them were analyzed according to age. Statistical comparison was performed with the independent t-test for continuous variables, and *p* < 0.05 was considered statistically significant.

Statistical analyses were performed by using different statistical software programs (STATA 13, StataCorp LLC, College Station, TX, USA).

## 3. Results

Among the 1179 women who performed DBTs at our department from January to April 2022, 104 were excluded (86 for BC surgery and 18 for breast augmentation with breast implants) (Figure 2).

The classification performed by radiologists and the Quantra software is presented in Table 2.

Among the 1075 women enrolled, aged between 40 and 86 years old (58.3 ± 7.2), radiologist n. 1 classified 61.2% as non-dense (A, B) and 38.8% as dense (C, D); radiologist n. 2 classified 58.1% and 41.9%, respectively; and radiologist n. 3 classified 54.4% and 45.6%, respectively. QDC was 51% for non-dense breasts (A: 11.5%, B: 39.5%), and 49% for dense breasts (C: 41.4%, D: 7.6%). Consensus between radiologists showed that of 1075 women, 626 (58.2%) were classified as having non-dense breasts, and 449 (41.8%) were classified as having dense breasts. In the age range 40–49 (46 ± 2.6), of 87 women, radiologist n. 1 classified 35.6% as non-dense breasts and 64.4% as dense breasts; radiologist n. 2 classified 25.3% and 74.7%, respectively; and radiologist n. 3 classified 23% and 77%, respectively. QDC was 19.5% for non-dense breasts (A: 3.4%, B: 16.1%), and 80.5% for dense breasts (C: 62.1%, D: 18.4%). Consensus among radiologists classified 24 women (27.6%) as non-dense and 63 (72.4%) as dense. In the age range 50–69 (58 ± 5.1), of 927 women, radiologist n. 1 classified 62.9% as non-dense breasts and 37.1% as dense breasts; radiologist n. 2 classified 60.2% and 39.8%, respectively; and radiologist n. 3 classified 56.5% and 43.5%, respectively. QDC was 53% for non-dense breasts (A: 12.3%, B: 40.7%), and 47% for dense breasts (C: 40.1%, D: 6.9%). Consensus among radiologists classified 559 (60.3%) as non-dense and 368 (39.7%) as dense. In the age range >70 (74 ± 4.2), of 61 women, radiologist n. 1 classified 72.2% as non-dense breasts and 27.8% as dense breasts; radiologist n. 2 classified 73.8% and 26.2%, respectively; and radiologist n. 3 classified 67.2% and 32.8%, respectively. QDC was 65.6% for non-dense breasts (A: 9.8%, B: 55.7%) and 34.4% for dense breasts (C: 31.1%, D: 3.3%). Consensus among radiologists classified 43 (70.5%) as non-dense and 18 (29.5%) as dense.

The agreement on breast density categories between radiologists and the Quantra software is reported in Table 3.

In particular, the agreement on breast density categories ranged from substantial to almost perfect between radiologists 1 and 2 (0.69–0.82) and radiologists 2 and 3 (0.77–0.83), with the highest agreement being in the screening age range (0.82 and 0.83, respectively). The agreement was substantial between radiologists 1 and 3 (0.63–0.78), with the highest agreement in the screening age range and the lowest in the over-70 age range (0.78 and 0.63, respectively). When compared to QDC, the agreement between each radiologist and the Quantra was heterogeneous, ranging from moderate (0.44) to substantial (0.78). When considering the consensus between radiologists compared to QDC, the agreement was moderate for the >70 age range and substantial for other age ranges.

Considering the two main broader categories of dense and non-dense breasts, the agreement between radiologists and the Quantra is reported in Table 4.

In particular, the agreement between radiologists ranged from substantial (0.70) to almost perfect (0.88), with the best agreement being in the screening age range. When compared to QDC, the agreement between each radiologist and Quantra ranged equally from substantial (0.61) to almost perfect (0.86), with the best agreement in the 40–49 and 50–69 age ranges for radiologists 2 and 3. Radiologist 1 had a lower value of agreement with QDC, ranging from 0.61 to 0.77.

When considering the consensus between radiologists, compared to QDC, the agreement was almost perfect for the screening age range (0.83), and substantial for the other two age ranges (0.74–0.78). There was a statistically significant difference in the distribution of breast density (McNemar’s test, *p* < 0.05) in the whole population and in the two age ranges of 40–49 and 50–69. (Table 3).

Out of the 1075 examinations, considering the four breast categories, 861 (80.1%) were concordant and 214 (19.9%) were discordant between the visual assessment consensus and the Quantra software. Considering the broader category of dense and non-dense breasts, 983 (91.4%) were concordant and 92 (8.6%) were discordant. According to the subjects’ age, no statistically significant difference was found between the concordant and discordant groups (*t*-test, *p* < 0.05) (Table 5).

## 4. Discussion

Visual assessment has demonstrated a strong association between density estimation and breast cancer risk; however, it suffers from subjective variability that limits its reproducibility [14].

Consequently, quantitative area-based methods have been developed. They can be classified into semi-automated (such as Cumulus and Madena), and fully automated (such as LIBRA, MedDensity, AutoDensity, and iReveal) [12]. These are based on the projected breast area, do not consider breast thickness, and require the radiologist to set segmentation thresholds by manually delineating the edge of the breast and the edge of the dense area to calculate the percentage of the dense breast. These extra-time- and effort-consuming steps limited their clinical applicability [24,25]. Therefore, over the years, methods for automated volumetric measurement of breast density have been developed. Currently, two automated volumetric density measurement tools are commercially available (i.e., the Volpara software and the Quantra software). In our study, we used the Quantra software to analyze the DBT images.

According to the BI-RADS 4th Edition, the Quantra software determined and reported the ratio of fibroglandular tissues to the total breast volume by using X-ray attenuations on the raw image in order to create an estimation of dense and non-dense tissue volumes for each pixel from the two volume estimates [26]. The volume ratio produced a volumetric fraction of fibroglandular tissue in percentages, referred to as breast volume density [27]. The results of Quantra BI-RADS 4th Edition categories 1–4 relate to an established reference population. In particular, the dense and non-dense breast tissues were nearly equally distributed within the general screening population, with 10% almost entirely fatty, 40% scattered fibroglandular, 40% heterogeneously dense, and 10% extremely dense [6]. Nevertheless, the exact cut-point thresholds between dense and non-dense breasts were never specified [28,29]. Several studies [30,31,32,33] have attempted to establish a percentage cutoff using the Quantra software to accurately stratify densities into high and low risk categories but have reported partly discordant or otherwise different results. Indeed, values ranged from 10% to 22% with great disagreement.

The BI-RADS 5th Edition has revised the mammographic breast density categories by excluding percentage quartiles for each of the four density categories in order to emphasize the texture descriptions of breast density, which reflect the masking effect of dense fibroglandular tissue on the mammographic depiction of noncalcified lesions. Consequently, the actual version of the Quantra software (2.2.3), through a proprietary algorithm powered by machine learning, analyzes mammography images for the distribution and texture of breast tissues, delivering a patient-specific breast density assessment. This version no longer displayed the volumetric density and associated parameters. The elimination of numerical breast density parameters from the display was a mandatory requirement by the FDA for a BI-RADS 5th Edition compliant product [22]. As part of the evidence supporting the approval of the Quantra 2.2 software in the US according to FDA guidelines, a study has been conducted to compare the estimation and consensus of radiologists’ assessments against each other and against the Quantra results. These data showed that the percentage of cases in which the two radiologists agreed on the BI-RADS assessment ranged from 63% to 86%, with an average of 76% [22]. Other studies [27,34] have been conducted to evaluate the inter-reader agreement and the concordance with automatic breast density assessment using the BI-RADS 5th Edition. Most of them showed a good correlation between automated and visual assessment. Nevertheless, a higher proportion of dense breasts were classified when using BI-RADS 5th Edition instead of 4th Edition.

According to current literature, our results showed a heterogeneous range of agreement between radiologists’ visual assessment and QDC, which is more frequently substantial, especially in the screening age range. The lower rate of agreement reported in the other two age ranges was probably due to the small sample size and the worst agreement between radiologists when classifying younger and older women. Indeed, as reported in the literature [35], a more subjective assessment system might change the distribution of assigned density categories, and more mammograms might be categorized toward dense breast tissues when there are localized dense tissues that would have been considered non-dense breast tissues according to the percentage quartile assessment. Nevertheless, Gastounioti A. et al. [15] showed an overall trend of downgrading breast density with visual assessment when imaging is performed with DBT. To our knowledge, only one study [21] has evaluated the automated evaluation of breast densities on DBT images.

In our study, the distribution of non-dense and dense breasts by consensus of radiologists showed a prevalence for categories A and B (16.6% and 41.6%, respectively) in comparison with categories C and D (35.8% and 6%, respectively). The concordance between the consensus of radiologists and QDC was substantial, as Quantra classified 11.4% as A, 39.5% as B, 41.4% as C, and 7.6% as D. This result reflects the trend depicted by Gastounioti and is consistent with the perception of less fibroglandular tissue in the volumetric display of DBT imaging compared with that of planar, area-based density in digital mammography alone.

Comparing the assessments of two main broader categories, different from what is reported in the literature, the agreement was almost perfect for the screening age range without a statistically significant difference between concordant and discordant cases when comparing them by age. The lack of statistically significant differences between concordant and discordant cases means that the two categories are very similar and that the assessment of radiologists and QDC is consistent and not influenced by any other factors. Nevertheless, it is important to note that even though the agreement is almost perfect, it may also be possible that the study does not have enough statistical power to detect small but clinically meaningful differences between the two categories due to the small BC sample size.

However, our population was mainly constituted of women aged between 50 and 69 years old (86.2%) in comparison to the younger (40–49 y, 8.1%) and older (>70 y, 5.7%). This made our results interesting since they were obtained by evaluating mostly the screening age range in which breast cancer is more frequent. Considering the different age ranges, the distribution of non-dense and dense breasts reported in Table 2 confirmed the progressive reduction of breast density with age. All these aspects are fundamental if breast density is universally recognized as an independent risk factor for the development of BC, as well as if breast density notification is introduced in some countries [36]. Furthermore, even though there is no clear association between breast density and breast cancer-specific survival [37,38], a high breast density might lead to missed detection and thus a later stage at diagnosis, when tumors are harder to treat. Consequently, a BC that is not screen-detected in women with high-density breasts may present as an interval cancer during the time between routine screenings. Accordingly, a correct evaluation of breast density, in association with the use of DBT, may modify the recall rate and the time interval between rounds, supporting an anticipation of diagnosis, especially during these challenging times when women have already suffered from a delay in diagnosis due to the consequences of the COVID-19 pandemic on BC screening [39].

## 5. Conclusions

Standardizing breast density evaluation can help radiologists to get involved in breast cancer screening by modifying the recall rate and personalizing screening procedures.

Since 50–69-year-old women constitute the majority of our population, our results were interesting as they might support the usefulness of breast density assessment in the target age range of breast cancer screening programs. Furthermore, the concordance between visual and automatic assessments in the dense and non-dense breast categories is an interesting result, supporting the importance of DBT in mitigating the masking effect of dense breasts and allowing the visualization of small breast cancers.

This study has some limitations. First, this is a single-institution study. All the mammograms were obtained from a single mammographic unit, and only the Quantra software was available in our institution. Furthermore, it was performed by three radiologists with a same workplace but different levels of experience. This may have led to similar assessments based on shared practice patterns. Furthermore, the small sample size of the 40–49 and over 70-year-old age ranges might limit the significance of the results for the assessment in these ranges. Lastly, the results of a consensus group might be biased if readers specifically focus only on mammographic density rather than on the detection of abnormalities in a real clinical setting.

Further evaluations are needed to make our results general and valid, but in our experience, the categorization proposed by the Quantra software has shown good agreement with the radiological evaluations, supporting the validity of human work. The recalibration of automated measurements is still in progress since it did not completely reflect the visual assessment; thus, clinical decisions regarding supplemental screening should be based on the radiologist’s perceived masking effect rather than the data produced by the Quantra software.

## Figures and Tables

**Table 1:** Kappa values.

Kappa Values	Type of Agreement
0.00–0.20	Slight
0.21–0.40	Fair
0.41–0.60	Moderate
0.61–0.80	Substantial
0.81–1.00	Almost perfect

Interpretation of weighted kappa values according to Landis J.R. and Koch G.G.

**Table 2:** Classification of breast density performed by radiologists and the Quantra software (QDC).

Density Category	Radiologists			Consensus	QDC
	1	2	3		
Total (1075)					
A	166 (15.4%)	163 (15.1%)	218 (20.3%)	179 (16.6%)	123 (11.5%)
B	492 (45.8%)	462 (43%)	367 (34.1%)	447 (41.6%)	425 (39.5%)
Non-dense	658 (61.2%)	625 (58.1%)	585 (54.4%)	626 (58.2%)	548 (51%)
C	354 (32.9%)	392 (36.5%)	409 (38.1%)	385 (35.8%)	445 (41.4%)
D	63 (5.9%)	58 (5.4%)	81 (7.5%)	64 (6%)	82 (7.6%)
Dense	417 (38.8%)	450 (41.9%)	490 (45.6%)	449 (41.8%)	527 (49%)
40–49 (87)					
A	4 (4.6%)	4 (4.6%)	5 (5.8%)	3 (3.5%)	3 (3.4%)
B	27 (31%)	18 (20.7%)	15 (17.2%)	21 (24.1%)	14 (16.1%)
Non-dense	31 (35.6%)	22 (25.3%)	20 (23%)	24 (27.6%)	17 (19.5%)
C	41 (47.1%)	54 (62.1%)	53 (60.9%)	50 (57.5%)	54 (62.1%)
D	15 (17.3%)	11 (12.6%)	14 (16.1%)	13 (14.9%)	16 (18.4%)
Dense	56 (64.4%)	65 (74.7%)	67 (77%)	63 (72.4%)	70 (80.5%)
50–69 (997)					
A	150 (16.2%)	148 (16%)	196 (21.1%)	164 (17.7%)	114 (12.3%)
B	433 (46.7%)	410 (44.2%)	328 (35.4%)	395 (42.6%)	377 (40.7%)
Non-dense	583 (62.9%)	558 (60.2%)	524 (56.5%)	559 (60.3%)	491 (53%)
C	297 (32%)	323 (34.8%)	338 (36.5%)	318 (34.3%)	372 (40.1%)
D	47 (5.1%)	46 (5%)	65 (7.0%)	50 (5.4%)	64 (6.9%)
Dense	344 (37.1%)	369 (39.8%)	403 (43.5%)	368 (39.7%)	436 (47%)
>70 (61)					
A	12 (19.7%)	11 (18%)	17 (27.9%)	12 (19.7%)	6 (9.8%)
B	32 (52.5%)	34 (55.8%)	24 (39.3%)	31 (50.8%)	34 (55.8%)
Non-dense	44 (72.2%)	45 (73.8%)	41 (67.2%)	43 (70.5%)	40 (65.6%)
C	16 (26.2%)	15 (24.6%)	18 (29.5%)	17 (27.9%)	19 (31.1%)
D	1 (1.6%)	1 (1.6%)	2 (3.3%)	1 (1.6%)	2 (3.3%)
Dense	17 (27.8%)	16 (26.2%)	20 (32.8%)	18 (29.5%)	21 (34.4%)

**Table 3:** K statistics. The agreement on breast density categories between radiologists (Rad1, Rad2, and Rad3) and the Quantra software (QDC). CI: 95% confidence interval. CON: consensus of the three radiologists.

Observers	OVERALL		40–49		50–69		>70	
	K	CI	K	CI	K	CI	K	CI
Rad1/Rad2	0.82	(0.77–0.86)	0.78	(0.64–0.92)	0.82	(0.78–0.87)	0.69	(0.51–0.87)
Rad1/Rad3	0.77	(0.73–0.82)	0.70	(0.56–0.84)	0.78	(0.74–0.83)	0.63	(0.45–0.81)
Rad2/Rad3	0.83	(0.79–0.87)	0.77	(0.63–0.91)	0.83	(0.78–0.87)	0.78	(0.61–0.96)
Rad1/QDC	0.69	(0.65–0.73)	0.65	(0.51–0.78)	0.70	(0.66–0.75)	0.44	(0.27–0.62)
Rad2/QDC	0.77	(0.73–0.81)	0.72	(0.58–0.86)	0.78	(0.73–0.82)	0.57	(0.39–0.74)
Rad3/QDC	0.78	(0.74–0.82)	0.76	(0.62–0.90)	0.78	(0.73–0.82)	0.65	(0.48–0.81)
CON/QDC	0.77	(0.73–0.81)	0.74	(0.60–0.88)	0.77	(0.73–0.82)	0.60	(0.43–0.78)

**Table 4:** K statistics. The agreement on the breast density category of dense/non-dense between radiologists (Rad1, Rad2, and Rad3) and the Quantra software (QDC). CI: 95% confidence interval. CON: consensus of the three radiologists. A *p*-value < 0.05 was considered statistically significant.

Observers	OVERALL			40–49			50–69			>70		
	K	CI	*p*	K	CI	*p*	K	CI	*p*	K	CI	*p*
Rad1/Rad2	0.87	(0.81–0.93)	<0.05	0.76	(0.56–0.96)	<0.05	0.88	(0.82–0.94)	<0.05	0.79	(0.54–1.04)	0.65
Rad1/Rad3	0.81	(0.75–0.87)	<0.05	0.70	(0.50–0.90)	<0.05	0.81	(0.75–0.88)	<0.05	0.81	(0.56–1.06)	<0.5
Rad2/Rad3	0.88	(0.82–0.94)	<0.05	0.81	(0.60–1.02)	<0.5	0.88	(0.82–0.94)	<0.05	0.84	(0.60–1.09)	<0.05
Rad1/QDC	0.76	(0.70–0.82)	<0.05	0.61	(0.42–0.80)	<0.05	0.77	(0.71–0.84)	<0.05	0.62	(0.37–0.87)	<0.5
Rad2/QDC	0.82	(0.76–0.88)	<0.05	0.84	(0.63–1.04)	<0.05	0.82	(0.76–0.88)	<0.05	0.65	(0.41–0.90)	<0.5
Rad3/QDC	0.86	(0.80–0.92)	<0.05	0.83	(0.62–1.04)	<0.5	0.86	(0.80–0.93)	<0.05	0.74	(0.49–0.99)	0.7
CON/QDC	0.83	(0.77–0.89)	<0.05	0.78	(0.57–0.98)	<0.05	0.83	(0.77–0.89)	<0.05	0.74	(0.49–0.99)	<0.5

**Table 5:** Concordant and discordant cases according to the four BI-RADS categories and the two main categories of dense and non-dense breasts. A *p*-value < 0.05 was considered statistically significant. SD: Standard Deviation.

Results	BI-RADS Categories		Dense—Non Dense Categories	
	N (%)	Average age ± SD	N (%)	Average age ± SD
Concordant	861 (80.1%)	58.1 ± 7.1	983	58.3 ± 7.1
Discordant	214 (19.1%)	59.0 ± 7.3	92	58.5 ± 7.3
*p* value	0.09		0.82	

## Data Availability

The dataset can be found on saniarp.it, in the Caserta LHA reporting database, and in the register of our daily activities.
